# Implementing a text-messaging intervention for unhealthy alcohol use in emergency departments: protocol for implementation strategy development and a pilot cluster randomized implementation trial

**DOI:** 10.1186/s43058-022-00333-y

**Published:** 2022-08-06

**Authors:** Megan A. O’Grady, Sandeep Kapoor, Laura Harrison, Nancy Kwon, Adekemi O. Suleiman, Frederick J. Muench

**Affiliations:** 1 Department of Public Health Sciences, School of Medicine, University of Connecticut, 263 Farmington Ave, Farmington, CT 06030-6325 USA; 2 Northwell Health, 350 Community Drive, Manhasset, NY 11030 USA; 3 Zucker School of Medicine at Hofstra/Northwell, 500 Hofstra Blvd, Hempstead, NY 11549 USA; 4 Partnership to End Addiction, 711 Third Avenue, 5th Floor, Suite 500, New York, NY 10017 USA

**Keywords:** Unhealthy alcohol use, Text-messaging intervention, Intervention mapping, Emergency department, Implementation barriers, i-PARIHS framework, Cluster randomized trial, Mixed methods

## Abstract

**Background:**

Unhealthy alcohol use (UAU) is a leading cause of premature mortality among adults in the USA. Emergency departments (EDs) are key intervention settings for UAU but often have limited time and resources. One low-burden, scalable approach to address UAU is text-messaging interventions. Despite strong research support and promise for scalability, there is little research on how to implement such interventions in healthcare settings. The process of providers making them available to patients in an efficient way within already busy and overburdened ED workflows and patients adopting them remains a new area of research. The purpose of this three-phase study is to develop and test an implementation strategy for UAU text-messaging interventions in EDs.

**Method:**

Our first aim is to examine barriers and facilitators to staff offering and patients accepting a text-messaging intervention in the ED using an explanatory, sequential mixed methods approach. We will examine alcohol screening data in the electronic health records of 17 EDs within a large integrated health system in the Northeast and conduct surveys among chairpersons in each. This data will be used to purposively sample 4 EDs for semi-structured interviews among 20 clinical staff, 20 patients, and 4 chairpersons. Our second aim is to conduct a stakeholder-engaged intervention mapping process to develop a multi-component implementation strategy for EDs. Our third aim is to conduct a mixed method 2-arm cluster randomized pilot study in 4 EDs that serve ~11,000 UAU patients per year to assess the feasibility, acceptability, and preliminary effectiveness of the implementation strategy. The Integrated Promoting Action on Research Implementation in Health Services framework will guide study activities.

**Discussion:**

Low-burden technology, like text messaging, along with targeted implementation support and strategies driven by identified barriers and facilitators could sustain large-scale ED-based alcohol screening programs and provide much needed support to patients who screen positive while reducing burden on EDs. The proposed study would be the first to develop and test this targeted implementation strategy and will prepare for a larger, fully powered hybrid effectiveness-implementation trial. Findings may also be broadly applicable to implementation of patient-facing mobile health technologies.

**Trial registration:**

This study was registered at ClinicalTrials.gov (NCT05350878) on 4/28/2022.

Contributions to the literature

- Emergency departments are important venues for addressing unhealthy alcohol use; however, given the limited time and resources, low-burden interventions are needed in this setting.
- Text-messaging interventions are low burden and potentially scalable in emergency departments to address unhealthy alcohol use, but limited research has examined their implementation.
- This study will examine barriers and facilitators to implementation of text-messaging interventions for unhealthy alcohol use in emergency departments and develop and test a multi-component implementation strategy.
- Study findings may be broadly applicable to the implementation of patient-facing mobile health interventions for substance use in ED settings—an understudied research area.

## Background

Unhealthy alcohol use (UAU) is estimated to cost the USA over $200 billion per year [1] and is one of the leading causes of premature mortality among adults [2, 3]. UAU is defined as a continuum of behaviors from risky or harmful use (exceeding recommended daily, weekly, or per occasion amounts) to alcohol use disorder [2]. National data suggests that, of the over 140 million current alcohol users, 47% exceeded recommended per occasion drinking amounts [4]. An estimated 14.5 million adults meet criteria for alcohol use disorder [4]. The prevalence of UAU was increasing among adults prior to the COVID-19 pandemic and the pandemic has further exacerbated UAU and related negative consequences [2, 5–7].

Emergency department (ED) visits involving alcohol consumption have increased in recent years [8] and UAU is more common among ED patients than in the general population [9]. It has been posited that substance use is the most important modifiable health behavior in the ED, [10, 11] as such the ED has been highlighted as a key intervention setting for UAU [12, 13]. Health systems are being tasked now more than ever with addressing substance use among their patients but often have limited time and resources [10]. ED clinicians work in high-volume, high acuity settings with significant time constraints and little training in providing intervention for substance use problems [10]. The ED is a promising point of intervention, yet scalable supports that reach as many patients with UAU as possible are needed.

Leveraging simple and efficient technology to support behavioral health intervention implementation in EDs has been urged [10, 14, 15]. The vast majority of ED patients (90%) express a strong preference for receiving health interventions via technology [15]. Access to mobile technology among ED patients who use substances is high: 97% report cell phone usage and many report a preference for technology-based means of receiving information about substance use [10]. Text messages are one of the most basic modes of mobile health interventions and use brief, supportive electronic messages transmitted via a mobile phone network to promote behavior change and may be the lowest burden, most preferred mobile health format [16–18]. Over the past decade, text-capable mobile phones have become ubiquitous across socioeconomic classes [19, 20]. It has been posited that text-message interventions have the potential to reduce health disparities at low cost because they appeal to the most underserved communities, including those that do not connect with traditional healthcare services [21]. Texts cost a fraction of a penny to send and unlimited texting plans are common [18, 22]. Interventions using text messages are low burden in that receiving intervention messages requires no user effort. They do not require logging in, tunneling through web pages, time-consuming data entry fields, or downloading an app [23]. Other advantages include being able to provide support over time in an individual’s natural environment and adapting content based on changing circumstances and feedback from the recipient [23]. From the health system perspective, text-messaging programs are relatively inexpensive to develop and maintain, do not require formal design, are agnostic to operating system, and expensive updates are not needed [18]. Text-based interventions may be the most feasible option for ED patient populations given the challenges of providing face-to-face intervention in EDs and they have the potential to reach traditionally underserved populations [15].

Reviews of text-messaging interventions for health behaviors broadly have found that the majority were effective and that there is strong evidence to support the value of integrating them into public health practice [18, 23, 24]. Text interventions are an acceptable and feasible strategy to enhance the delivery of care for persons with substance use problems [17, 25–27]. A recent meta-analysis found that text-messaging alcohol interventions reduce alcohol consumption compared with no or basic health information [28]. For example, people with harmful drinking patterns significantly reduced drinking frequency and quantity after receiving daily texts [29]. Patients with alcohol use disorder perceive that text interventions may help them remain abstinent [30]. The benefits of harnessing text interventions for UAU in the ED could be considerable because they are low burden for patients and acceptable and effective [31]. For example, discharged trauma center patients who received a text intervention reduced hazardous drinking as compared to usual care controls [32] and the majority of trauma patients would enroll if offered a text intervention [33]. Among patients recruited from EDs, text interventions for UAU are effective in supporting them in meeting drinking goals and making drinking reductions, including reductions in binge drinking and number of drinks consumed per day [34, 35]. Furthermore, 44% of ED patients accepted a text intervention and those who accepted had higher rates of treatment attendance after discharge from the ED as compared to those who did not accept [9]. These studies show broad efficacy for text interventions in EDs with potential to reach diverse populations; however, no studies have developed and tested a strategy for systematically offering them to patients in EDs in a scalable manner.

Systematic reviews have noted that very few studies examine implementation and dissemination of mobile health technology [10, 36, 37]. Recent failures of pragmatic mobile health trials due to lack of patient and staff uptake are a warning that careful implementation approaches are needed [38, 39]. A complex web of inter-related technical, social, patient, and organizational considerations may be at play [40–42]; yet insufficient guidance is available to inform larger scale implementation of mobile health [10, 12, 15, 17, 19, 23, 43–45]. Two recent reviews of substance use technology interventions noted that only one study [46] focused explicitly on implementation outcomes; most focused only on patient clinical outcomes [37, 47]. Few of these studies mention implementation strategies, making it difficult to know how to successfully implement technology into practice [37]. Other reviews urge the field to move toward developing text intervention implementation strategies [17, 48], with one stating that “the field awaits true dissemination and implementation studies in which text interventions are put into place in real world settings.” [18]. Research should prioritize examining techniques for increasing uptake of alcohol text interventions among patients as well as examining whether ED staff are likely to systematically offer such interventions [9].

Implementation of text interventions for UAU will require EDs to address patient, setting, administrative, and staff barriers. This study will use the Integrated Promoting Action on Research Implementation in Health Services (i-PARIHS) framework to guide intervention and research activities [49, 50]. i-PARIHS posits that optimal implementation occurs when practice facilitation promotes the acceptance and use of a new practice innovation based on both the recipient’s needs and on the unique nature of the inner and outer context [50, 51]. i-PARIHS positions facilitation as the active ingredient to help navigate individuals and teams through complex change processes by addressing (a) the innovation’s degree of fit within the existing practice; (b) the motivations, beliefs, characteristics, and resources of the intervention recipients; and (c) the inner and outer context (e.g., leadership support, culture, the learning environment, organizational priorities, capacity for change, mandates). Facilitation has been defined as interactive problem solving that is active, dynamic, and task-oriented and has been used in a number of disciplines [50, 52, 53]. Notably, facilitation has been utilized for several decades and has now become routine for implementing changes [54–56]. ED-based studies have started to use facilitation along with comprehensive implementation strategies because typical strategies such as educational sessions or grand rounds presentations are not enough to implement new practices successfully [57]. However, i-PARIHS does not dictate specific implementation strategies given that each context, recipient, and intervention may call for a different set of strategies. Selecting implementation strategies is a complex task and strategies are too often selected in an unsystematic way, fail to address key contextual determinants, and are not well matched to the contexts in which they are deployed [58]. Rigorous methods are needed to select strategies that take into account relevant theory and stakeholder participation and that are specified clearly enough to be replicated [59, 60]. This study will use a systematic intervention mapping process to develop the facilitated implementation strategy [59, 61–64]. The aims of this study are to:

Aim 1: examine potential barriers and facilitators to staff offering and patients accepting a text-messaging intervention in the ED using an explanatory, sequential mixed methods approach

Aim 2: use a stakeholder-engaged intervention mapping process to develop a multi-component implementation strategy for EDs

Aim 3: conduct a mixed method 2-arm cluster randomized pilot study in 4 EDs that serve ~11,000 UAU patients per year to assess the feasibility, acceptability, and preliminary effectiveness of the implementation strategy

## Methods

### Aim 1: Identify barriers and facilitators to implementing a text-messaging intervention for UAU in EDs

#### Design

The study will be conducted within a large integrated health system in the Northeastern United States that has 17 EDs serving adult patients. For aim 1, we will use an explanatory, sequential mixed methods approach that places more emphasis on the qualitative data (quant → QUAL) [65] given that little is known about barriers/facilitators to text-messaging intervention implementation in EDs. First, we will examine alcohol screening data in the electronic health records (EHRs) of the 17 EDs and conduct surveys among chairpersons in each. This data will then be used to purposively sample 4 EDs for semi-structured interviews among clinical staff (e.g., nurses, physicians), patients, and chairpersons (2 with low perceived implementation barriers; 2 with high perceived barriers).

#### Quantitative data sources, sample, and analysis

All EDs in this study use the structured, validated 3-item AUDIT-C screening tool [66] to detect UAU. It is built into their EHRs which capture screening results and reasons for screening deferral if applicable (e.g., acuity of illness, including mental status and intoxication) or patient refusal. Data on alcohol screening from the past 12 months will be extracted and compiled from the EHRs of the 17 EDs to include: % patients screened (numerator: # AUDIT-C screens completed; denominator: total patient census) and % screenings deferred (numerator: # patients with AUDIT-C screening deferral indicated; denominator: total patient census). EHR data on alcohol screening is being examined because it provides an important clue to each ED’s buy-in and ability to identify and address UAU. For example, EDs with low screening completion rates may indicate staff hesitancy, lack of time to screen patients, or an EHR where the AUDIT-C has no reminder prompt for completion. Such factors may limit ED’s ability to properly identify appropriate patients and offer them a text intervention. ED chairpersons (or their designee) of each of the 17 EDs will complete a 12-item online survey measuring their views on the feasibility, acceptability, and appropriateness of implementing a text-messaging intervention in EDs that is adapted from a validated measure [67]. We will calculate an average score for each chairperson.

#### Qualitative data sources, sample, and analysis

We will identify 4 ED sites (2 with low perceived implementation barriers and 2 with high perceived barriers) for semi-structured interviews using stratified purposeful sampling [68, 69]. We will create low and high categories based on ED alcohol screening completion rates (low = ≤ median; high = > median) and survey score (low = score ≤ median; high = above the median).

##### ED chairperson and staff

In each of the 4 selected EDs, chairpersons who completed the quantitative survey will be interviewed. Modeled on other implementation studies [70], we will then ask each chairperson to identify 5 clinical staff for interviews (e.g., nurses, physicians, support staff). Interviews (~30 min) will be conducted over the phone or in-person depending on staff preference. If conducted over the phone, we will follow best practices for phone-based qualitative interviews in health services research [71, 72]. Interview guides for staff, informed by the i-PARIHS framework [57, 73], will cover views on alcohol screening in the ED, text-messaging interventions and how to best offer them to patients, and barriers/facilitators to intervention implementation and general practice change.

##### ED patients

To recruit patients for interviews, following procedures from similar ED studies [10], patients will be approached in the ED if they screened positive on the AUDIT-C (≥3) as identified in the EHR. We will recruit 5 patients from each of the 4 EDs. Interviews (~30 min) will be conducted in a private area in the ED and patients will be offered a $15 gift card. If saturation is not reached with the initial samples, additional interviews will be conducted. Interview guides for patients will be adapted from a study that assessed patients’ preferences and attitudes toward technology interventions initiated in the ED [15] and a semi-structured interview guide used among trauma patients on this topic [33] (e.g., mobile device ownership and use, interest in receiving supportive messages via mobile device on UAU reduction, best ways to offer patients the intervention in the ED, concerns about using mobile devices). Expert consultants in implementation science and ED-based text messaging will provide feedback on interview guides.

##### Qualitative analysis

Interviews will be recorded and transcribed then analyzed using directed content analysis [74]. We will use a deductive approach such that a codebook with codes and operational definitions will be created prior to analysis using key elements from i-PARIHS [75]. A two-person coding team, led by the first author, will be used. We will use Atlas.ti to manage, organize, and examine patterns in the data and will follow recommended steps for content analysis [75, 76]: (1) transcripts will be read several times by coding team, (2) meaning units within the transcripts will be identified (i.e., smallest amount of text that contains needed insights) [75], (3) meaning units will be coded; data that cannot be coded using initial codes will be identified and analyzed after initial coding to determine if they represent a new category or sub-category of an existing code [74], (4) codes will be sorted into categories, and (5) themes will be formed by grouping two or more categories together. Coding disagreements will be resolved through discussion. We will use a continuous process of coding, categorizing, and reviewing the raw data to reflect on the analysis at various points and make revisions (e.g., recoding data). Weekly coding meetings will be held to review coding decisions, discuss discrepancies, and check progress. We will use several methods to increase trustworthiness [77]: sample to the point of saturation, conduct negative case analysis, use member checking, keep an audit trail on study decisions, maintain a detailed codebook, and track inter-coder reliability.

### Aim 2: Develop a stakeholder-driven multi-component strategy for implementation of a text-messaging intervention for UAU in the ED

We will use a systematic 4-step intervention mapping method [59, 61–63] to develop our implementation strategy. We will leverage aim 1 results and three stakeholder meetings. Stakeholder meetings will consist of ED chairpersons, administrators, staff, and patients from within the integrated health system where the study is being conducted as well as ED stakeholders external to the health system. The intervention mapping approach will create a transparent, deliberate, stakeholder-engaged process while aligning with strategies and terminology in the implementation science literature.

#### Intervention mapping: step 1

The first step is a needs assessment. The study team will compile results from aim 1 quantitative and qualitative data to assess implementation needs.

#### Intervention mapping: step 2

Aim 1 results will be presented to a stakeholder group consisting of ED staff, chairpersons, administrators, patients, and expert consultants to identify concrete objectives and expected outcomes of the implementation strategy. For example, the group will identify what would need to be changed at the setting, patient, staff, and administrative levels for successful implementation of the text intervention. The study team will create a matrix of needs, goals, and objectives for successful implementation resulting from this meeting.

#### Intervention mapping: step 3

**Fig. 1:**
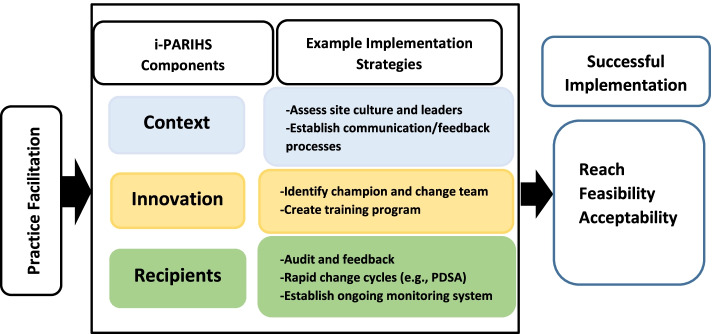
The i-PARIHS model and example implementation strategies and outcomes

This step involves identifying theory-based, practical discrete implementation strategies to meet the needs, goals, and objectives from step 2. The study team will select implementation strategies with the following principles in mind: (1) appropriateness for the ED setting, (2) replicability and generalizability, (3) feasibility and sustainability for future dissemination, and (4) alignment with the i-PARIHS components to address ED needs, constraints, resources, and context. Implementation strategies will be selected from the i-PARIHS facilitator’s toolkit [49] and other expert-consolidated implementation strategy lists [78] and the following will be identified for each implementation strategy chosen: active ingredient, causal mechanisms by which they will exert the desired changes, mode of delivery (e.g., face to face), and the intended target (e.g., administrators, front-line staff, patients) [79]. See Fig. 1 for sample implementation strategies mapped onto the i-PARIHS framework. Guided by the i-PARIHS framework, we will use practice facilitation, and an internal-external facilitator team will support sites’ use of the identified implementation strategies. Internal-external facilitation is used commonly [80, 81] and leverages internal provider motivation with outside expertise and program support [50, 82]. The second stakeholder meeting will be used to receive feedback on and refine the selected implementation strategies.

#### Intervention mapping: step 4

The multi-component implementation strategy will be designed, and its execution planned in the final step. The strategies identified in step 3 will be operationalized to clearly delineate what they entail, as well as how they will be delivered. Materials (e.g., manuals, training materials) to support the implementation strategy will also be created. The third stakeholder meeting will be held to get feedback on the execution plan and materials.

### Aim 3: Examine the feasibility, acceptability, and preliminary effectiveness of the implementation strategy

#### Design

In line with recommendations pertaining to the scope and target outcomes of pilot studies for implementation trials [83–85], the main goal of this pilot trial is to assess the (1) feasibility (i.e., perceptions among ED clinical staff members that the implementation strategy can be successfully used), (2) acceptability (i.e., perceptions among ED clinical staff members that the implementation strategy is agreeable, palatable, or satisfactory), and (3) and preliminary effectiveness of the implementation strategy. We will operationalize our effectiveness outcome as “reach” (i.e., proportion of eligible patients that enroll in the text intervention), which is a key outcome recommended in the implementation science literature [83, 85–87]. The design is a mixed method, two-armed, study in 4 EDs (2 assigned to the new implementation strategy vs. 2 implementation as usual control). Qualitative and quantitative measures will be collected and analyzed simultaneously and merged—qualitative data will provide context to and explanation of the quantitative results in a complimentary way, representing a QUAL + QUAN structure that gives equal emphasis to both types of data [65].

#### Study site randomization

The study will be conducted within a large integrated health system in the Northeast of the USA. Because the number of study sites for this pilot is relatively small, simple unrestricted randomization may not be sufficient to ensure balance between study conditions. Pairing of EDs would improve balance but may reduce study power and is not recommended for small numbers of clusters [88]. Therefore, we will use a minimization procedure to ensure overall balance on three important covariates: annual ED census, location (urban/suburban), and AUDIT-C positive rate. A computerized algorithm will be used to identify all possible allocations that meet the balancing constraints and one of the allocations will be randomly selected [89]. A data analyst not associated with the study will conduct this procedure to minimize bias. Study sites and data analysts will be blinded to the study condition.

#### Study conditions

##### Implementation strategy condition

Through virtual and in-person meetings, the internal/external facilitation team will support sites in utilizing the implementation strategy developed in aim 2 for 7 months: 2 months to prepare policies/workflows for offering the text intervention to patients and 5 months actively supporting implementation of offering the text intervention.

##### Control condition

In control “implementation as usual” sites, no facilitation or implementation strategies will be provided. Controls will receive (1) an informational session on the text intervention during grand rounds/staff meetings and (2) flyers to provide patients with intervention enrollment information. In both conditions, it will be emphasized that any patient who screens positive on the AUDIT-C (as per site protocol in the EHR) are appropriate [34, 35].

#### Text-messaging intervention

The text-messaging intervention to be implemented in this pilot trial [29] includes daily texts tailored to individuals’ responses to brief enrollment and check-in assessments (e.g., drinking goals, self-efficacy). Just-in-time support is also provided (e.g., text “temp” for support to manage a craving to drink). An RCT testing this intervention found 94% retention of participants over 12 weeks, and reductions in the number of heavy drinking days in groups that received active messages (vs. assessment only), with the greatest effect for just-in-time adaptive tailoring. To enroll in the intervention, participants text a number (e.g., REDUCE to 55753) and then receive a welcome message that will prompt them to complete the enrollment assessment. We selected this particular texting intervention for implementation in this study because in a recent meta-analysis it was the only study that had positive outcomes and met all criteria for low bias [90]. Furthermore, it has three key ingredients recommended by the broad text-messaging intervention literature: theory-driven, evidence-based, and adaptively tailored.

#### Data sources and measures

##### Effectiveness/reach

Effectiveness will be examined by calculating how many patients were eligible (positive AUDIT-C) based on EHR data and the proportion who enrolled in the intervention as collected by the text platform. Demographics will also be collected from the EHR and text platform to examine differences in characteristics between who was eligible based on AUDIT-C score and who enrolled. In the text platform, each study site will have a unique enrollment text code number, allowing for tracking of patients that enrolled by site and when they enrolled, demographics, and responses to the intervention tailoring questions (e.g., drinking patterns, self-efficacy, motivation). We anticipate there will be ~5000 UAU eligible patients identified during the study trial period based on (1) sites’ current census, (2) current screen positive rates, and (3) screening completion rates based on our previous evaluations in this integrated health system.

##### Feasibility and acceptability

Feasibility and acceptability of the implementation strategy among staff in the 2 intervention sites will be measured by two 4-item validated measures: the Acceptability of Intervention Measure (AIM), and Feasibility of Intervention Measure (FIM) (e.g., the implementation strategy seems easy to use; the implementation strategy meets my approval) [67]. We will also adapt these measures to assess patients’ views on feasibility and acceptability of the text-messaging intervention to provide context to the effectiveness/reach outcomes.

##### Qualitative measures

Semi-structured interview guides for staff will be developed using concepts from the Organizational Readiness to Change Assessment, an instrument designed to assess the core elements of the i-PARIHS model, as well the aim 1 interview guide (e.g., What was it like to offer the text interventions to patients?) [91]. Semi-structured interview guides for patients will be adapted from a previous text-messaging study [92] in order to provide context to the reach/effectiveness data (e.g., Describe how the intervention was offered to you? What was it like for you to receive messages? What aspects of the text messaging, if any, do you think helped/did not help? What made you interested in enrolling in the text-messaging program?).

#### Data collection procedures

##### Patients

A random sample (*n* = 250) of adult patients who screen positive for UAU and enroll in the text intervention will be invited via text message to participate in a survey on the intervention’s feasibility and acceptability 1 month after enrolling in the intervention. A link will be sent to participants via the text-messaging platform for them to participate in the online survey, review informed consent study information sheet, and receive a $15 e-gift card for their participation.

We will also invite 10 patients to participate in qualitative interviews from each of the 4 EDs with a question at the end of the survey inquiring if they are interested; we will then follow up by phone to schedule. A verbal consenting procedure will be conducted by the interviewer along with an information sheet provided to the patient.

##### Staff

Clinical staff members at the 2 intervention EDs will be invited by email to participate in an online survey; during month 6 of the trial, they will receive a study information sheet informing them of their rights as a research participant. One chairperson per site will also be interviewed with the semi-structured interview guide. We will ask chairpersons to identify 20 staff per intervention ED (*n* = 40) to participate in semi-structured interviews, targeting those with knowledge of the intervention implementation. Staff and patients will have the option to complete the interview in-person or over the phone; interviews will be recorded. Staff will receive a study information sheet and a verbal consenting procedure will be used. Additional interviews will be conducted if our sample does not reach saturation [77].

#### Analysis

The same procedures described in aim 1 will be used to analyze qualitative data for aim 3. For the reach/effectiveness quantitative data, we will calculate a proportion for each study condition (e.g., numerator: # of patients in control or intervention sites who enrolled in the intervention; denominator: # of patients with positive AUDIT-C) and use *z*-tests for independent proportions [93] to examine whether control and intervention sites differ significantly. We will also use *z*-tests to examine differences in demographics between those eligible and enrolled to determine whether there are gender or racial disparities. For these proportional analyses [94], to achieve 80% power, a sample size of at least 300 in each condition is needed (total of 600) to detect a 10% difference in proportions with significance level of *p* = .05. For the feasibility/acceptability staff and patient survey data, we will calculate descriptive statistics for each. We will triangulate this quantitative data with the qualitative data by merging the results. No identifying information will be maintained by the study team for any staff or patients; all participants will be identified with a participant ID.

## Discussion

EDs are under pressure to provide services for UAU and are potentially significant points of intervention for UAU. Text-messaging interventions have the potential to be a scalable solution that could be used by almost any ED patient and may better support underserved populations. However, little is understood about how to ensure that ED staff systematically offer these products to patients nor how to best engage patients with them during an ED visit. This study will develop and test a comprehensive set of implementation strategies for text-messaging alcohol interventions under “real world” conditions. We will use a deliberative stakeholder-guided intervention mapping procedure to develop the implementation strategy. While applied widely to development of public health interventions, this intervention mapping approach is less frequently used to develop implementation strategies but offers great potential to align the selected implementation strategies and terminology in the implementation science literature, making the strategy more easily replicable for others wishing to use the approach.

In terms of future implications of study findings, as recommended in Pearson et al. [83], the research team and stakeholder group will set a priori criteria to determine whether to progress to a larger scale, fully powered hybrid implementation-effectiveness trial based on preliminary implementation strategy effectiveness in conjunction with feasibility and acceptability [94, 95]. Trial results will be shared with the stakeholder group as well as at scientific conferences and in scientific publications. This larger scale test, if warranted, would expand the number of EDs and the range of implementation outcomes beyond “reach” as well as examine cost-effectiveness of the implementation strategy and effectiveness of the text-messaging intervention in reducing patient alcohol use.

The focus of this study is primarily on studying implementation outcomes, and not patient outcomes; therefore, we will not randomize patients to the text-messaging intervention; instead, all patients will receive the intervention. However, we will collect valuable data from patients to assess feasibility and acceptability of the intervention from their perspective; determine the proportion who engage with the intervention in a real-world situation; examine disparities in gender, race, and ethnicity in enrolled vs. not; and understand why patients enrolled. All of the more than 150 hospitals in New York State are now required by state regulation to screen for substance misuse and such policies are being implemented elsewhere [96]; therefore, findings have the potential to be applied state- and nation-wide and a large number of sites would be available for a larger study.

This project moves forward the implementation science on technology interventions, a fairly new area of research. While the technology focus of this project is on text messaging, findings may be broadly applicable to patient-facing mHealth technologies. Text-messaging interventions have the potential to reach as many patients impacted by UAU as possible, including those traditionally underserved, in a scalable, low-burden manner; this study will illuminate strategies to reach that potential.

## Data Availability

Not currently applicable. Data from the pilot trial will be available in the NIAAA data sharing archive: https://nda.nih.gov/niaaa/.

## Abbreviations

AUDIT-C: Alcohol Use Disorders Identification Test-Consumption
ED: Emergency department
EHR: Electronic health record
i-PARIHS: Integrated Promoting Action on Research Implementation in Health Services
UAU: Unhealthy alcohol use
